# Low-dose radiation research insights in experimental animals: A gateway to therapeutic implications

**DOI:** 10.14202/vetworld.2024.2253-2258

**Published:** 2024-10-07

**Authors:** Nayanatara Arunkumar, Megha Gokul, Harini Narayanam, A. K. Ananya

**Affiliations:** 1 Department of Physiology, Kasturba Medical College Mangalore, Manipal Academy of Higher Education, Karnataka, Manipal, 576104, India; 2 Department of Physiology, Manipal University College Malaysia, Melaka, Malacca, Malaysia; 3 Srinivas Institute of Medical Sciences and Research Center, Mangaluru, Karnataka, India

**Keywords:** animal model, high dose radiation, low dose radiation, mice, rat

## Abstract

In recent years, a significant research effort has been underway to explore the effects of low-dose radiation (LDR). Animal models play a key role in various fields of research, including biomedicine, pharmaceutical, environmental, and behavioral studies. The use of animal models has been an invaluable tool in radiation research for understanding radiation biology, assessing radiation risks, and developing strategies for radiation protection and medical management. In the present review, the initial part focuses on the deleterious effects of high-dose radiation, and in correlation to that, in the later part of the review, the emphasis has been given to experimental approaches to explore the beneficial effects of LDR using animal models. This review could help explore the innovative approach for future research targeting the therapeutic role of LDR in various diseases, including depression, Cancer, Parkinson’s disease, and Alzheimer’s disease.

## Introduction

The concept of hormesis holds that different systems in the body can be stimulated or react favorably to decreased concentrations of an agent that is harmful at larger doses during physical or biological exposure. The linear-no-threshold hypothesis neglects that the evolution of life and development on earth has been exposed to natural background radiation [1]. Physiological effects at low doses cannot be predicted by assessing harmful results observed at high doses [2]. Thus, the notion that all biological systems can react favorably to exposure to low levels of ionizing radiation signifies the theoretical concept of radiation hormesis [3]. It is the phenomenon where higher doses of ionizing radiation cause deleterious effects, whereas low-dose radiation (LDR) causes stimulatory effects [4]. The use of animal models has helped explore the present knowledge about the biological consequences of different intensities of radiation exposure. Even though there is a good source of data from human exposure, the interpersonal heterogeneity in the human population is significantly lower. Preliminary animal studies have helped in understanding the concepts in LDR. The conclusions of these data are based on published scientific observations from animal experiments, radiation sources, and cells exposed to radiation. The conclusion from these observations requires supporting evidence from animal studies. Research data indicating the harmful effect of high-dose radiation has been well explored in many studies [5, 6], but the beneficial effect of LDR using animal models has been a leading research topic in recent years.

This review highlights insight into various innovative research studies that explore the beneficial role of LDR using animal models, which can help in future therapeutic approaches in clinical scenarios.

For the current review, an extensive literature survey was conducted using PubMed, Scopus, and Web of Science databases. Importance was given to studies on the effects of LDR. Our search was focused on research experiments that discussed the outcomes of LDR exposure in various animal models. Parallelly, we also investigated studies that assess the effect of exposure to high-dose radiation. The resulting matches were then screened for duplicates, and the abstracts and titles were then manually screened and sorted according to the inclusion criteria involving only articles discussing the impact of radiation sources of high- and LDR.

## Basic Concepts of Radiation

Radiation is usually used in two categories: diagnostic and therapeutic. Depending on the purpose, ionizing radiation is administered through internal or external sources [7]. Various medical conditions require the use of ionizing radiation in clinical procedures, each involving differing levels of radiation exposure [5, 6]. In clinical settings, the therapeutic benefits of ionizing radiation play an important role in the modern world. X-ray imaging, computed tomography, magnetic resonance imaging, and nuclear medicine are in significant demand [5]. In radiation oncology and teletherapy, ionizing radiation is used to treat patients with cancer using combinations of chemotherapy and surgical treatments [6]. These techniques are used in different stages of cancer. The primary treatment is pre-surgery, post-surgery, or advanced and late stages of cancer. Brachytherapy and nuclear medicine involve the internal administration of radiation sources [8]. In brachytherapy, radiation is exposed by a minute source implanted directly in the tumor tissue or body cavities. This minute source emits ionizing radiation that kills cancer cells. Therapeutic nuclear medicine involves the administration of a radionuclide in liquid form through oral or intravenous administration [9]. There are advanced techniques and procedures for both diagnostic and therapeutic irradiation to increase the efficiency of the target cell. The extent of damage to the surrounding cells of the tissues is very limited.

## Direct and Indirect effects of Ionizing Radiation

Ionizing radiation causes damage to biomolecules through two methods: the direct method, which passes through cells, and the atoms of the biomolecules, which get excited by gaining energy from ionizing radiation. It causes severe damage to vital cell components. The second indirect method involves the radiolysis of intercellular water molecules present in the cytoplasm that generate highly unstable free radicals. The indirect mechanism of early cell death occurs due to DNA damage in the early phase of cell division. In an indirect radiation-induced method, free radicals cause oxidation of lipids and proteins in the cell membrane. Different types of free radicals are produced by different mechanisms [10].

## Damaging effects of High-dose Radiation

Ionizing radiation must be utilized with utmost care and caution. The range of radiation doses depends on the modality of the disease. High-dose radiation has proven to be detrimental in various studies and scientific reports [6, 11]. High-dose radiation therapy is a standard option for treating cancer, during which damage to normal tissue occurs, causing genotoxicity and cytotoxicity [12]. Pre- and post-exposure to high-dose radiation documents the harmful effects of radiation on all organ systems of the body [13]. Rats exposed to 9 Gy died within 30 days [14] and minimum mortality was reported in animals exposed to 6 Gy [15]. A radiation dosage of 11 Gy in animals showed signs of sickness within 2–4 days [16]. Kumar *et al*. [17] reported that animals exposed to 8 Gy radiation showed a decrease in body weight after the 25^th^ day of radiation. The signals generated by damaged cells activate the repair pathway that maintains genomic integrity. Zhao *et al*. [18] reported a radiation-induced significant decrease in blood cells after irradiation at 4 and 8 Gy. Considering these reports, total blood counts have become a regular practice in all patients undergoing radiation therapy. The coding sequences of important genes may lead to neoplastic transformation of normal tissues that are unavoidably exposed.

High radiation levels can damage nucleic acids by affecting the permeability of neurovascular tissue. High-dose radiation leads to changes in the functional and morphological components of the brain. A recent meta-analysis of brain exposure to high-dose ionizing radiation demonstrated the release of neuroinflammatory markers [19]. Patients receiving high-dose radiation therapy experience a variety of side effects, including cognitive impairment, behavioral disturbances, and seizures [20]. Although the damage caused by high-dose radiation exposure to biological processes, molecular pathways, and cellular functions has been well explored and established, the effects of LDR levels on human health are still unclear. Less research has been published in the field of low-energy use than in high-energy use.

## LDR and its Beneficial Role

In daily life, human beings are unavoidably exposed to LDR from natural and human-made sources. Basic knowledge and a detailed understanding of the impact of low-dose ionizing radiation have become increasingly important because LDR is an emerging research area due to its therapeutic approach. The impact of LDR on cellular components has become increasingly important because of its increased use in modern medicine, research, industry, and security. Based on the literature, the classification of LDR includes dosages ranging from 0 to 100 mGy [21]. The average annual background radiation exposure to humans is approximately 3 mGy. Industrial applications and diagnostic equipment containing radioactive materials contribute to radiation exposure.

Various studies and scientific experiments have provided evidence of differences in biological responses to high and low radiation doses [22, 23]. A previous study by Cui *et al*. [24] provided evidence of the effects of radiation hormesis on cell survival, immune response, and cytogenetic protection. Several animal studies [21–24] have shown that LDR supports normal cell growth, promotes enzymatic and tissue repair, boosts immunity, and prevents aging. A study by Rosen *et al*. [16] also reported the effect of LDR in delaying cancer cell growth. The protective role of LDR against DNA repair mechanisms and anti-inflammatory responses has been reported.

Moreover, LDR stimulated each component of the protective antioxidant systems, reduced genomic instability, and prevented free radical-induced damage. LDR-induced hormesis combats cancer through various immune mechanisms. Furthermore, LDR-induced hormesis is associated with significant changes in cytokine and chemokine production. As established in the literature, increased stimulatory cytokines and decreased immunosuppressive cytokines promote cell proliferation, which might promote anticancer protection. The findings of Betlazar *et al*. [25] provided novel insights into the protective mechanisms of LDR, particularly its role in antioxidant defense within the rat brain. LDR augmented neurogenesis in the hippocampus, enhancing cognitive performance and promoting cognitive health [25]. Another study by Yahyapour *et al*. [26] involving a mouse model of Parkinson’s disease showed that LDR reduced oxidative stress and apoptosis in affected neurons. These results suggest the protective role of LDR against Parkinson’s disease. The effects of LDR exposure on the central nervous system are consistent with radiation hormesis. A few studies show that LDR promotes repair mechanisms against pathological mechanisms in LDR. Yin *et al*. [27], in their experiments using mouse brains, suggested that 0.1 Gy radiation was neuroprotective by inducing alterations in gene expression. LDR also causes microglial activation that mediates the immune response [28]. The beneficial roles of LDR in a wide variety of microbes, plants, invertebrates, and vertebrates have been explored [29]. Marples *et al*. [30] showed that LDR reduced Alzheimer’s-related brain plaques in mice. Various clinicians and researchers have been investigating the effects of LDR, suggesting its utility as a therapeutic tool for several neurodegenerative diseases.

LDR exposure stabilized transcription and translational processes [31]. Research in a mouse model indicated that LDR increased antioxidant levels in immune organs, detoxified reactive oxidation species, and repaired DNA damage [32]. LDR decreased the neoplastic transformation frequency below the spontaneous rate. It activated the immune response, suppressed metastasis and spontaneous cancers, and significantly increased plasma calcium concentrations in Klotho gene mutant mice [33]. Low-linear energy transfer X-ray radiation caused a notable shift in offspring coat color toward pseudo-agouti and resulted in methylation differences between experimental and control mice of the same coat color class during the first 7 days of gestation in agouti viable yellow (Avy) mice [34]. Epigenetic changes following LDR in the Avy mouse model contributed to radiation hormesis [35].

Additionally, epigenetic modifications and various signal transduction pathways, such as Ataxia Telangiectasia Mutated (ATM), Extracellular Signal-Regulated Kinase (ERK), p38 Mitogen-Activated Protein Kinase (MRPK), c-Jun N-terminal Kinase (JNK), Tumor Protein P53 (P53), and gene expression, have been implicated in LDR-induced benefits [4]. In the mouse immune system, LDR-activated lymphocytes, through signals from antigen-presenting cells, lead to increased surface molecule concentrations, reduced cyclic adenosine monophosphate/cyclic guanosine monophosphate ratios, and suppression of the phospholipase A2-prostaglandin E2 pathway [35]. LDR-induced upregulation of the antioxidative gene peroxiredoxin-2 in the retina could offer a new therapeutic approach for retinitis pigmentosa and other neurodegenerative diseases [36]. Chronic low-dose exposure in rats did not significantly increase stroke symptoms [37]. Further study in these areas may provide valuable insights into the relationship between LDR and circulatory diseases. LDR improved cognitive function and reduced amyloid plaque accumulation in advanced AD stages in 5xFAD mice [37]. Research from various groups also indicated that low-dose ionizing radiation could help reduce arthritic changes in multiple animal models, which is similar to pharmaceutical agents [38, 39]. In mice with the pink-eyed unstable mutation, exposure to LDR during pregnancy can result in genetic reversions in the mouse embryo [40].

Furthermore, 10 mGy gamma radiation was observed to boost the expression of transformation-related proteins in liver and spleen tissue [41]. In pKZ1 mouse prostate tissue, very low radiation doses ranging from 0.005 to 0.01 mGy were noted to induce chromosomal inversions, whereas doses of 1 and 10 mGy reduced inversions compared with untreated samples [41, 42]. These results suggest that the pKZ1 transgene is a reliable marker for detecting responses to prolonged exposure to low radiation levels. More research is needed to explore the health implications of genetic and epigenetic alterations. LDR boosts the immune response by enhancing the reactive response of T cells to mitogenic stimulation, altering cytokine release and immune cell populations, and enhancing T cell activation capacity by dendritic cells [24]. Subsequent research has focused on the effects of LDR on autoimmune diseases. Studies have shown that the impact of low-dose γ-irradiation varies depending on the type of immunological disease. It can either attenuate or intensify pathologies, depending on the source of the antigen causing the disease. More in-depth research is needed to explore the effects of LDR as a therapy for immunological diseases.

In recent years, significant efforts have been dedicated to developing new strategies to prevent and mitigate radiation’s harmful effects on tissues, including the brain. These novel strategies include radiation shielding, Fractionated radiation therapy, and the administration of radioprotective agents before or during radiation therapy. These strategies balance the need for effective radiation treatment while minimizing its adverse effects on the brain. Radiation exposure negatively affects the dendritic space, impairs the brain’s ability to generate new neurons, and leads to behavioral changes. Consequently, mitigation strategies should focus on restoring these essential functions to preserve cognitive health and overall brain function. Smith and Doe [43] analyzed the molecular, neuroanatomical, and behavioral impacts of LDR on the rodent brain, both directly and indirectly. Their study specifically examined the effects of scatter irradiation in an animal model. One rodent received direct liver irradiation, while another nearby rodent was shielded with medical-grade lead; however, the shielded rodent’s brain was still exposed to scatter irradiation. This setup allowed researchers to explore the indirect effects of radiation on the brain in a controlled environment. This study is the first to demonstrate that even very low-dose scatter irradiation can significantly affect gene expression, dendritic morphology, and behavior in exposed animals [43]. LDR induces cyclin D1 accumulation in the cytoplasm of human keratinocytes. Cyclin D1 is a key regulatory protein involved in cell cycle progression, particularly during the transition from G1 to S phase. Accumulation in the cytoplasm rather than the nucleus can influence various cellular processes, including apoptosis. The findings suggest that LDR influences cellular processes by modulating cyclin D1 localization, thereby affecting keratinocyte behavior and contributing to changes in cell survival and apoptosis [44, 45]. This understanding is crucial for developing strategies to manage radiation effects and improve therapeutic outcomes.

## Conclusion

**Figure-1:**
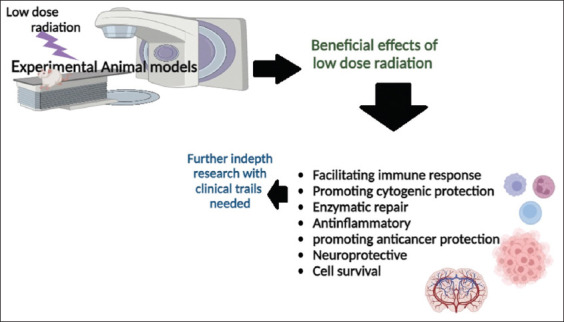
Beneficial role of low-dose radiation [Source: The figure was made by the authors using Biorender.com].

Research on LDR has demonstrated promising beneficial effects, including potential improvements in neurogenesis, cognitive function, and cellular protection (Figure-1). These findings indicate that LDR may offer a novel approach to treating and managing various disease conditions, including Alzheimer’s disease, Parkinson’s disease, Multiple sclerosis, stroke, inflammatory diseases, and psychiatric diseases, including depression. In the future, its efficacy and safety for these applications could be confirmed based on a molecular approach, including proteomics and genomics. This approach could help understand the mechanisms for assessing the practical applications of LDR in clinical settings. In the future, the insights gained from this research could pave the way for innovative therapeutic strategies that positively impact health, technology, and the environment.

## Limitations

Despite these promising outcomes, several limitations must be acknowledged. Variability in experimental conditions and methodologies can also affect the reproducibility and generalizability of findings. Furthermore, the mechanisms through which LDR influences cell processes remain complex and require further detailed analysis.

## Future Scope of Research

Preclinical animal models can lay the groundwork for developing biomarkers for LDR, offering valuable insights into its effects. The beneficial effects of LDR research could lead to significant advancements in clinical approaches. The significant effects observed through various studies suggest that LDR could be a novel treatment approach for various disease conditions. As researchers delve deeper into understanding the impacts of LDR, society stands to gain invaluable insights that could positively impact health, technology, and the environment in the future. Future research should focus on several key areas to address the current limitations and expand the understanding of LDR. Comprehensive studies using diverse and well-characterized animal models, including neurodegenerative, diabetic, and epileptic models, are necessary to validate the observed biopositive effects. More studies are needed to explore LDR’s long-term safety and efficacy to determine its suitability for clinical application. Developing and validating biomarkers for LDR are crucial for assessing its effects and optimizing therapeutic protocols. In addition, investigating the molecular mechanisms and pathways affected by LDR could help to enhance the ability to harness its benefits while minimizing potential risks. Clinical trials will also be essential for proving these findings into practical and effective treatments for chronic diseases, which could further support evidence for the development of cheap and effective therapeutic approaches in the future.

## Authors’ Contributions

NA: Drafted the manuscript. MG, HN, and AKA: Data acquisition and reviewed the manuscript. All authors have read, reviewed, and approved the final version of the manuscript.
